# Supplementary material for: Generalizability of Deep Learning Models for Caries Detection in Near-Infrared Light Transillumination Images
Source: J Clin Med. 2021 Mar 1;10(5):961. doi: 10.3390/jcm10050961 (PMC7957685; doi:10.3390/jcm10050961)
Supplement: Supplementary file 1 [file jcm-10-00961-s001.pdf]

Journal of
Clinical Medicine

Table S1. Batch size and learning rate resulting in the best performing models for the four scenarios discussed.

	train/test in vitro	train/test in vivo	train in vitro/test in vivo	train in vivo/test in vitro
batch size	16	8	8	8
learning rate	5e-6	5e-5	5e-5	5e-5
